# A statistical evaluation of the sexual dimorphism of the acetabulum in an Iberian population

**DOI:** 10.1007/s00414-024-03334-9

**Published:** 2024-09-27

**Authors:** Varsha Warrier, Marta San-Millán

**Affiliations:** 1 School of Sciences, College of Science and Engineering, University of Derby, Derby, UK; 2 Medical Sciences Department, Clinical Anatomy, Embriology and Neuroscience Research Group (NEOMA), Faculty of Medicine, University of Girona, Girona, Spain

**Keywords:** Forensic anthropology, Human identification, Sex estimation, Acetabulum, Discriminant analysis, Logistic regression, Machine learning, Accuracy

## Abstract

Sex estimation is essential for human identification within bioarchaeological and medico-legal contexts. Amongst the sexually dimorphic skeletal elements commonly utilised for this purpose, the pelvis is usually preferred because of its direct relationship with reproduction. Furthermore, the posterior part of the innominate bone has proven to have better preservation within degraded contexts. With the aim of investigating the potential of the vertical acetabular diameter as a sex marker, 668 documented individuals from three different Iberian skeletal collections were randomly divided into training and test samples and eventually analysed using different statistical approaches. Two traditional (Discriminant Function Analysis and Logistic Regression Analysis) and four Machine learning methodologies (Support Vector Classification, Decision Tree Classification, k Nearest Neighbour Classification, and Neural Networks) were performed and compared. Amongst these statistical modalities, Machine Learning methodologies yielded better accuracy outcomes, with DTC garnering highest accuracy percentages of 83.59% and 89.85% with the sex-pooled and female samples, respectively. With males, ANN yielded highest accuracy percentage of 87.70%, when compared to other statistical approaches. Higher accuracy obtained with ML, along with its minimal statistical assumptions, warrant these approaches to be increasingly utilised for further investigations involving sex estimation and human identification. In this line, the creation of a statistical platform with easier user interface can render such robust statistical modalities accessible to researchers and practitioners, effectively maximising its practical use. Future investigations should attempt to achieve this goal, alongside examining the influence of factors such as age, on the obtained accuracy outcomes.

## Introduction

Sex estimation constitutes one of the fundamental analyses that is undertaken for human identification within bioarchaeological, paleodemographic, and medico-legal contexts [1, 2]. Sexing from the human skeleton has often been attempted through inspectional and/or metric examinations of different bony markers. While macroscopic inspectional examinations allow for a quicker analysis, these often present with certain pertinent drawbacks. In addition to warranting a greater degree of training and expertise, morphological visual assessments suffer from a higher degree of subjectivity [2]. Osteometric methods, on the other hand, confer greater objectivity to the process of human identification, and thus are being increasingly preferred [3–7].

Within the human skeleton, innominate-based markers are often preferred for sex estimation [8, 9], attributable to its biological association with reproduction which manifests as variations in rates and direction of growth of local areas [10], which in turn confers high accuracy for sexing [1, 11]. Amongst the various sex markers within the human pelvis, the acetabulum presents as a reliable [4, 12], and taphonomically resilient structure [13]. Morphoscopic and osteometric attributes of the acetabulum have previously been utilised for sex estimation [2–4, 12, 14–34], with a recent predominance of the latter. Acetabular diameters have already been considered as potential sexually dimorphic variables, in isolation [4, 12, 23]. However, previously undertaken multivariable investigations have demonstrated that the acetabular diameter is one of the least accurate sexing parameters, when compared to other pelvic variables, within a worldwide comprehensive dry bone sample [8, 9]. Certain other authors, by contrast, concluded that the vertical acetabular diameter was the most reliable variable out of the nine pelvic parameters analysed in a CT-based Spanish sample [35]. In addition to these differential findings, there also exists a certain methodological variability between these investigations. The former study defined the vertical acetabular diameter following Braüer [36], while the latter considered it differently under the definition given by Genovés [37]. Such contrasting methodological approaches and empirical findings warrant further investigations into the sexing accuracy associated with the vertical acetabular diameter, and the need for standardisation. Furthermore, similar investigations with acetabular dimensions on dry bone are currently wanting for an Iberian population.

In recent times, statistical analysis has been increasingly incorporated into morphological and osteometric sex estimation. Such statistical integrations, in addition to enhancing sex prediction, aid in computing the probability of an individual/ skeletal remain being biologically male or female. A large share of sex estimation-based studies have often utilised Discriminant Function Analysis (DFA) and Logistic Regression (LR) for sexing [38–42]. Sexing with the acetabulum has, by and large, also been undertaken using DFA [2, 4, 12, 15–20, 23–25, 43, 44]. Probabilistic Sex Diagnosis (DSP) has also been utilised for sexing the innominate [8, 9, 28–31, 34]. Machine Learning Algorithms present as another equally viable statistical modality for sexing. Machine Learning (ML) offers numerous advantages within the domain of forensic identification. In addition to circumventing the requirement for a normally distributed sample, ML has fewer statistical assumptions to satisfy, can supplement missing values, and effectively do away with the Measurements Statistics Controversy [27]. However, despite the applicability of ML approaches for sex estimation, as indicated by investigations with other anatomical markers [45–49], its specific use for sexing the acetabulum is largely unexplored. Furthermore, investigations comparing the accuracy percentages obtained with different statistical approaches for sexing the acetabulum, are also presently lacking.

In light of the lacuna associated with acetabular sexing, the present study was designed with the following objectives: (a) establishing the sexing accuracy of the vertical acetabular diameter for an Iberian population, and (b) estimating the highest accuracy achievable with the acetabular diameter for this population through the use of different statistical approaches.

## Materials and methods

### Sample

668 documented individuals (321 females and 347 males) from three modern documented skeletal collections from the Iberian peninsula were assessed for this study: (1) UVA - Valladolid collection [50], housed in the Anatomical Museum at the Faculty of Medicine of the University of Valladolid (Valladolid, Spain); (2) UCM - Madrid collection [51], housed in the School of Legal Medicine at the Faculty of Medicine of the University Complutense of Madrid (Madrid, Spain); and (3) MBL - Luis Lopes collection [52], housed in the Museo Bocage of Lisbon (National Museum of Natural History, Lisbon, Portugal). All these collections are derived from modern cemeteries and have documented records of birth and death. All three collections comprise of individuals who died in the 20th century, except for five specimens from the Lisbon repository. This comprehensive sample was chosen as it is representative of different biogeographical affinities of the Iberian population, their availability, and their potentially large age ranges.

Individuals with mature acetabula (completely fused) were selected in order to cover the entire period of acetabular maturity. For Iberian populations, the complete fusion of the acetabulum has been reported to transpire at 15 years in males and at 12 years in females [17, 53, 54]. This pattern is in accordance with the acetabular maturity standard intervals published by Schaefer and colleagues [55] i.e., 14–18 years for males, and 11–16 years for females. For the present study, considering the previous inclusion criteria, individuals older than 15 years with fused acetabulum were chosen in order to create the same age intervals in both sexes, giving an effective age range of 15 to 98 years. Table 1 displays information regarding age, sex, and collection of the individuals analysed in this study. The *left os coxa*, without any pathology and/or deformity that might affect the analysis, was chosen for each individual. In cases where the left acetabulum was missing, or broken (i.e., where the positioning of the sliding calliper on the acetabular rim was not possible), or pathologically impacted, the right counterpart was analysed (16.86% of the total sample).

**Table 1:** Descriptive parameters for each skeletal collection, grouped by sex (*N* = 668)

Collection	Sex	*N* (%)	Age range (years)	Mean ± SD (years)
UVA	F	49 (07.33)	37–96	73.31 ± 14.241
	M	69 (10.32)	23–88	64.12 ± 14.802
UCM	F	74 (11.07)	21–97	64.53 ± 19.160
	M	96 (14.37)	20–94	53.03 ± 18.454
MBL	F	198 (29.64)	15–98	54.13 ± 22.005
	M	182 (27.24)	15–89	51.26 ± 21.529

UVA = University of Valladolid collection; UCM = University Complutense of Madrid collection; MBL = Museo Bocage of Lisbon; M = male; F = female; SD = standard deviation

The final study sample comprised of 668 skeletal remains: 118 from the UVA collection (mean age ± SD = 68.79 ± 15.158 years), 170 from the UCM collection (mean age ± SD = 58.00 ± 19.559 years), and 380 from the MBL collection (mean age ± SD = 52.76 ± 21.797 years) (Table 1). Documented age information for one female of the UCM collection was unavailable within the study sample.

### Measuring the acetabular diameter

**Fig. 1:**
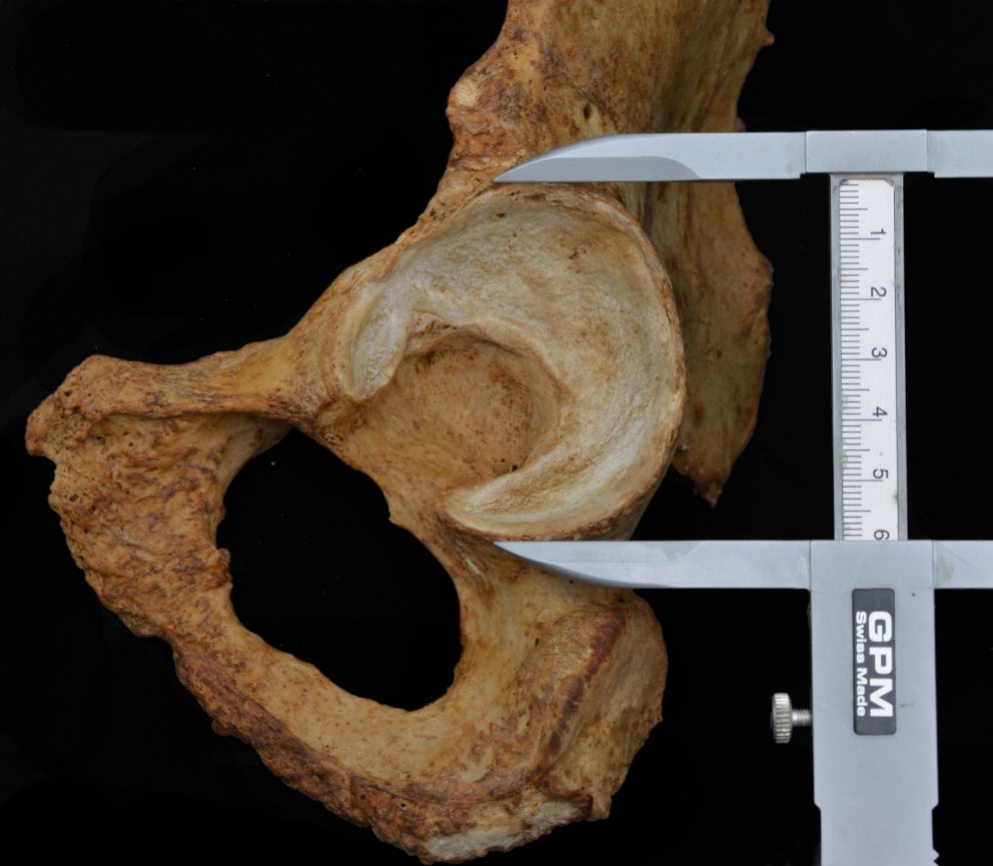
Vertical acetabular diameter measured on the acetabular rim, parallel to the long axis of the ischial body [56] by a standard calliper

The maximum vertical diameter of the acetabulum was measured on the acetabular rim, parallel to the long axis of the ischial body following the definition of Taylor and DiBennardo [56] (Fig. 1). The variable was measured with a standard sliding calliper up to one decimal place (centimetres).

### Statistical analyses

Statistical analyses were undertaken with IBM Statistical Package for Social Sciences (SPSS) v26 and Python 3. Statistical tests of normality, intra-observer reliability, sex differences, and between group differences were assessed using SPSS (see below). Accuracy for sex estimation was established using different statistical modalities of Discriminant Function Analysis, Logistic Regression Analysis, Support Vector Classification, Decision Tree Classification, k Nearest Neighbour Classification, and Neural Networks. While statistical models of Discriminant Function Analysis, Logistic Regression Analysis, Support Vector Classification, Decision Tree Classification, and k Nearest Neighbour Classification were derived with Python, Neural Networks were developed using SPSS. This bivariate statistical approach was utilised because when attempts were undertaken to derive neural networks with Python, the ‘*tf. losses.sparse_softmax_cross_entropy’* function was found to be deprecated, with a corresponding warning message displayed for the use of ‘TensorFlow’ for creating a neural network. To avoid obtaining inaccurate performance measures, alternatively, SPSS was utilised to create neural networks for sex estimation. A p-value < 0.05 was considered statistically significant for all computations.

Normality of the sample was tested using the Kolmogorov Smirnov test. Obtained results (*p* < 0.05) indicated a non-normal distribution of the sample, and thus non-parametric statistical tests were utilised during further analysis.

To analyse the intra-observer reliability associated with this dimension, acetabular diameter data from a previous study [57] from the School of Legal Medicine at the Faculty of Medicine of the Complutense University of Madrid (Madrid, Spain) [51] was used. In order to establish the intra-observer error, 108 left acetabular measurements (i.e. two sets of measurements obtained from the same documented skeletal remains, at an interval of three years) were utilised, and a two-way random intraclass correlation (ICC) was calculated. Due to the data characteristics, absolute agreement ICC type was used and as reported by [58], single measures were employed for intra-observer reliability. Obtained absolute agreement ICC values were interpreted following the system of Koo and Li [59], i.e., ICC values < 0.5 are indicative of poor reliability, values between 0.5 and 0.75 indicate moderate reliability, values between 0.75 and 0.9 indicate good reliability, and values > 0.90 indicate excellent reliability. Inter-observer analysis was not undertaken as the vertical acetabular diameter has already demonstrated acceptable levels of error between observers, in the past [8, 29, 33, 34, 60, 61].

Sexual dimorphism associated with the acetabular diameter was evaluated using the Mann-Whitney U test. Sex differences were assessed for the overall sample, as well as for the three collections individually. Coefficient of correlation between acetabular diameter and sex was estimated for the overall sample, as well as for the three individual collections.

Significant differences, if any, in acetabular diameter values between the three collections were assessed using the Kruskal-Wallis test. No significant differences were obtained between the three collections, and thus, further statistical analysis was undertaken with the pooled sample.

In order to compute the sexing accuracy associated with the acetabular diameter for an Iberian population, the entire study sample of 668 individuals was divided into a training group of 567 individuals, and a test group of 101 individuals, using the “scikit. learn” function in Python in an 85%- 15% proportion, and the “partitioning” function in SPSS, wherever relevant (see above section on bivariate approaches utilised for deriving sex estimation models). The training group was subjected to different statistical tests and was utilised to derive various sex estimation models (Linear Discriminant Function Analysis, Quadratic Discriminant Function Analysis, Logistic Regression Analysis, Artificial Neural Networks, Support Vector Classifier, CRT Decision Tree Classifier, and k Nearest Neighbour Classifier), while the test group was employed to validate these derived models.

### Discriminant function analysis (DFA)

Discriminant Function Analysis is often used to classify an unknown individual into one of the known reference groups (sex in this present study) through computations of the Mahalanobis distance [62]. There are two main variants of DFA; Linear Discriminant Function Analysis (LDFA) and Quadratic Discriminant Function Analysis (QDFA).

LDFA employs a linear combination of predictor variables and assumes equality of covariance. In LDFA, classification of an individual into known reference groups (sex) is undertaken as follows [63]:

$$y = {\rm{ }}{a_0} + {\rm{ }}\sum\limits_{{\rm{i = 1}}}^{\rm{m}} {{a_i}{X_i}}$$

Where Σ is the pooled sample covariance matrix, a_0_ and a_i_ are adjustable parameters that are calculated using linear/ multilinear log regression, and X_i_ is the independent variable.

QDFA, on the other hand, utilizes a non-linear combination of predictor variables and does not assume equal covariance. Classification of an individual into known reference groups occurs as follows [63–65]:

$${\rm{d}}_{\rm{k}}{\rm{(x)}}{\rm{=}}{\rm{-1/2}}{\rm{(x-\mu_{k})}}^{\rm{t}}\sum\limits_{\rm{k}}^{\rm{-1}}{\rm{(x-\mu_{k})}}  {\rm{-1/2\ln\mid}}{{\rm\Sigma}}_{\rm{k}}{\rm{\mid}}{\rm{\,+}}{\rm { \,ln}}{\rm {\,P}}_{\rm{k}}$$

where k (class) = 0/1 (female/ male), Σ_k_ is the covariance matrix for class k, P_k_ denotes the prior probabilities (prior probabilities are estimated as n_k_/n where n_k_ are the number of cases for class k, and n is the total number of cases), ½ (x-µ_k_) is the Mahalanobis distance between the unknown to the known reference group’s centroid. The above equation can also be re-written as [63]:

$${\rm{y = }}{{\rm{a}}_{\rm{0}}}{\rm{ + }}\sum\limits_{{\rm{i = 1}}}^{\rm{m}} {{{\rm{a}}_{\rm{i}}}{{\rm{X}}_{\rm{i}}}} {\rm{ + }}\sum\limits_{{\rm{i = 1}}}^{\rm{m}} {{{\rm{b}}_{\rm{i}}}{{\rm{X}}_{\rm{i}}}^{\rm{2}}} {{\rm{ + }}_{}}\sum\limits_{{\rm{i < j}}}^{\rm{m}} {{{\rm{c}}_{{\rm{ij}}}}{{\rm{X}}_{\rm{i}}}{{\rm{X}}_{\rm{j}}}}$$

Linear, as well as Quadratic Discriminant Function Analysis was undertaken in the present study, so as to not presume a linear relationship between diameter and sex. Demarking points were established by applying both Discriminant approaches to the training set. Accuracy percentages were computed for the total population, males, and females of the training group (*N* = 567) to, primarily, illustrate the sexing accuracy associated with the acetabular diameter for an Iberian population, as given below:

$$\eqalign{& {\rm{Accuracy = Number\,of\,individuals\,correctly\, sexed}} \cr & {\rm{/\,Total\,number\,of\,individuals}} \cr}$$

In order to enable further validation of DFA for sexing, accuracy percentages were estimated for the total population, males, and females of the test set (*N* = 101). In addition to this, Receiver Operating Characteristic (ROC) curves, a form of graphical representation of the performance of binary classifier models, were also plotted. ROC curves were plotted using both Discriminant approaches, for the test group to demonstrate the discriminatory ability of these statistical approaches.

### Logistic regression analysis (LRA)

Unlike DFA, which employs the use of the Mahalanobis distance to estimate sex, Logistic Regression Analysis calculates the probability of an individual belonging to a specific group (male/ female) [27]. Binary Logistic Regression (BLR) assumes a logistic distribution of errors, and establishes a relationship between the predictors of sex and the probability of an individual belonging to a particular sex class using the following expression [63]:

$${\rm{ln }}\left( {{\rm{P sex = k}}} \right){\rm{/ 1 - }}\left( {{\rm{P sex = k}}} \right){\rm{ = }}{{\rm{c}}_{\rm{0}}}{\rm{ + }}{{\rm{c}}_{\rm{1}}}{{\rm{X}}_{\rm{1}}}{\rm{ + }}{{\rm{c}}_{\rm{2}}}{{\rm{X}}_{\rm{2}}}{\rm{ + }}.......$$

where c_0_, c_1_, c_2_ etc. are adjustable parameters determined using a maximum likelihood ratio, X_1_, X_2_ etc. are the sex traits, and k is the sex class = 0/1 (female/ male) [63]. Logistic regression analysis was first undertaken with the training set in the present study, and accuracy was computed for the total population, males, and females. Derived LRA model was then applied to the test set and accuracy percentages for both sexes and the total population were estimated. An ROC curve was plotted to illustrate the discriminatory ability of LRA with the test set.

### Neural networks

An artificial neural network (ANN) is a system of interconnected neurons which mimics the human brain and classifies an individual into different classes (as either male, or female in the present study) [63, 66, 67]. A typical neural network consists of three layers; an input layer, a middle-hidden layer, and an output layer. Within every neural network, the input variables are transformed into the output through weights, as follows [63, 67]:

$${\rm{sex = f(}}{{\rm{W}}_{\left( {{\rm{m + 1}}} \right){\rm{ p + 1}}}}{\rm{ + }}\sum\limits_{{\rm{i = 1}}}^{\rm{p}} {{{\rm{W}}_{\left( {{\rm{m + 1}}} \right){\rm{ p + i}}}}{{\rm{H}}_{\rm{i}}}} {\rm{)}}$$

which can also be rewritten as:

$$H_{i}= f(W_{(i-1)(m+1){+1}} + \sum_{j=1}^{m}w_{(i-1)(m+1)+1\,j}X_{j})$$

where f is the activation function of the network, m is the number of input nodes, p is the number of nodes in the hidden layer, and w denotes the weights employed for transforming inputs to output.

An artificial neural network was created from the training set, with a single input and a standardised rescaling method for the sex predictor. Different hidden layers, and activation functions for the middle and output layers were utilised, so as to discern the best trained ANN model for sex estimation. Accuracy percentages were computed for males, females, and the total population of both, the training set and test set. ROC curves were plotted to establish the discriminatory power of ANN, with the test group.

### Support Vector classification (SVC)

Support Vector Machines (SVM) allow for regression and classification through its use of the maximum marginal hyperplane, non-linear transformations, and no prior distributional assumptions [68–70]. SVMs are particularly effective for binary classification problems. Support Vector Classification is used to classify an individual into one of the different classes (sex in this present study), by finding and constructing a separating hyperplane which maximises the margin between the instances of the two sexes [46], as:

$${{\rm{d}}_{\rm{H}}}{\rm{((}}{{\rm{x}}_{\rm{0}}}{\rm{)) = |}}{{\rm{w}}^{\rm{T}}}{\rm{((}}{{\rm{x}}_{\rm{0}}}{\rm{) + b}}\left| {{\rm{ / }}} \right|\left| {\rm{w}} \right|{{\rm{|}}_{\rm{2}}}$$

$${\rm{w* = ar}}{{\rm{g}}_{\rm{w}}}{\rm{max[mi}}{{\rm{n}}_{\rm{n}}}{{\rm{d}}_{\rm{H}}}{\rm{((}}{{\rm{x}}_{\rm{n}}}{\rm{))]}}$$

The hyperplane is then used to classify individuals of the test set as:

$${{\rm{y}}_{\rm{n}}}{\rm{[}}{{\rm{w}}^{\rm{T}}}\left( {\rm{x}} \right){\rm{ + b] = }}\left\{ \ge{{\rm{0\,if\,correct; < 0\,if\,incorrect}}} \right\}$$

where b is the intercept and bias term of the hyperplane equation, (x) is the given point vector, and ||w||_2_ is the Euclidean norm for the length.

The training set was utilised to train the SVC model, and accuracy obtained with sexing males, females, and the overall population of the training set was computed. Subsequently, the trained model was applied to the test set, and accuracy measures for males, females, and the total population were estimated for this smaller subset of 101 remains. An ROC curve was plotted with the test set to demonstrate the discriminating ability associated with SVC.

### Decision tree classification (DTC)

Decision trees are non-parametric machine learning approaches, commonly utilised for both, classification and regression problems. The hierarchal tree structure comprising of root nodes, branches, internal nodes, and leaf nodes is used to classify an individual into one of the two classes (sexes). Decision Tree Classification and the use of the Decision Tree Algorithm employs the use of different measures of impurity- Entropy and information gain, or Gini impurity (https://www.datascienceprophet.com/understanding-the-mathematics-behind-the-decision-tree-algorithm-part-i/*).*

Entropy is the amount of information required to accurately describe data. It is estimated as:

$${\rm{Entropy }}\left( {\rm{S}} \right){\rm{ = - }}\sum\limits_{{{\rm{c}}_{\rm{\varepsilon }}}{\rm{C}}} {{\rm{p}}\left( {\rm{c}} \right){\rm{lo}}{{\rm{g}}_{\rm{2}}}{\rm{p}}\left( {\rm{c}} \right)}$$

where S is the dataset, c represents the classes (i.e., sexes), p(c) represents the proportion of datapoints which belong to class c to the total number of datapoints. These entropy values fall between 0 and 1, and the predictor with lowest entropy value is utilised to split the tree. Information gain, which is further employed to create multiple nodes, does so as follows:

$$\eqalign{& {\rm{Information\,gain (S,a) = }} \cr & {\rm{Entropy }}\left( {\rm{S}} \right){\rm{ - }}\sum\limits_{{\rm{v}}{ \in ^{{\rm{vcalues(a)}}}}} {{\rm{|}}{{\rm{S}}_{\rm{v}}}\left| {{\rm{/ }}} \right|{\rm{S| Entropy (}}{{\rm{S}}_{\rm{v}}}{\rm{)}}} \cr}$$

where a represents the attribute or class label, |Sv|/ |S| denotes the proportion of values in Sv to those of S, Entropy (S) is the entropy of dataset S, and Entropy (Sv) is the entropy of dataset Sv.

Gini impurity, the other commonly employed measure of impurity, computes impurity in the node, as:

$${\rm{Gini\,impurity = 1 - }}{{\rm{\Sigma }}_{\rm{i}}}{{\rm{(}}{{\rm{p}}_{\rm{i}}}{\rm{)}}^{\rm{2}}}$$

Based on these measures of impurity and homogeneity, the decision tree is split accordingly.

Decision Trees were created from the training set using different growing methods, and accuracy was estimated for males, females, and the total population. The generated tree was subsequently validated using the same accuracy measures (i.e., for males, females, and the total population) for the test set. ROC curve was plotted for the test set and discriminatory power of the Decision Tree was assessed.

### k nearest neighbour classification (kNN)

kNN is a non-parametric supervised Machine Learning classifier which classifies an individual into different classes (sex) based on proximity. kNN algorithm is also a part of the “Lazy Learning” Machine Learning models, as is does not undergo a characteristic training phase. As a result, computation occurs when prediction is being made. In order to determine the points closest to the query point/ subject and subsequently classify it, distance metrics need to be computed. Distance metrics are often estimated through Euclidean distance, Manhattan distance, Minkowski distance, Hamming distance, amongst others. Amongst these, the most common is the Euclidean distance which estimates the distance between query point and closest points, as follows:

$${\rm{d}}\left( {{\rm{x,y}}} \right){\rm{ = }}\sqrt {\sum\limits_{{\rm{i = 1}}}^{\rm{n}} {{{{\rm{(}}{{\rm{y}}_{\rm{i}}}{\rm{- }}{{\rm{x}}_{\rm{i}}}{\rm{)}}}^{\rm{2}}}} }$$

Classification using the kNN algorithm also requires selecting the nearest neighbour which will be utilised for classification. Lower k values, however, can result in high variance and low bias, and high k values will lead to high bias and low variance.

kNN classification was undertaken with the training set, and accuracy was computed for males, females, and the total population. Accuracy values were similarly estimated for both sexes separately, as well as for the overall population of the test set. An ROC curve was additionally plotted using the test set to illustrate the discriminatory ability of kNN classification between the two biological sexes.

Accuracy values obtained using different statistical approaches were compared in order to establish the highest accuracy attainable with the acetabular diameter. These values were subsequently also comparatively scrutinised against those reported within existing literature.

## Results

Based on the classification mentioned in the Methods section, good reliability was achieved during intra-observer reliability test for the acetabular diameter [0.854 (*p* < 0.001)].

A Mann-Whitney U test yielded significant differences (Mann-Whitney U = 11762.000; *p* < 0.001) in acetabular diameter values between sexes for the entire collection of 668 remains, with males having a significantly higher value than females. Sexual dimorphism, when evaluated for each of three collections, indicated significant differences as well (Table 2). A Spearman’s rho of 0.683 was obtained between sex and acetabular diameter for the entire study sample. Corresponding correlations of 0.672, 0.716, 0.668 were obtained for the UVA, UCM, and MBL collections, respectively. All correlation values were statistically significant. In addition, amongst the three collections, highest values for acetabular diameter were obtained with females and males of the UCM collection (Table 2), but the differences with the other datasets were not statistically significant.

**Table 2:** Descriptive parameters for acetabular diameter for each skeletal collection, grouped by sex (*N* = 668)

Collection	Sex	Mean diameter ± SD (cm)	Diameter range (cm)	Mann-Whitney U	*p*-value
UVA	F	48.57 ± 2.84	43.00–55.00	4125.000	< 0.001
	M	54.43 ± 3.58	46.00–64.00		
UCM	F	49.22 ± 2.57	44.00–57.00	595.000	< 0.001
	M	55.26 ± 3.67	46.50–67.00		
MBL	F	49.17 ± 2.84	40.00–58.00	361.000	< 0.001
	M	54.40 ± 3.40	41.50–60.00		

UVA = University of Valladolid collection; UCM = University Complutense of Madrid collection; MBL = Museo Bocage of Lisbon; M = male; F = female; SD = standard deviation

### Characteristics of utilised statistical modalities

For DFA, Linear and Quadratic Discriminant Analysis, both, yielded a demarking point of 51.8 centimetres, and a coefficient of discriminant of 0.555. An intercept of -28.77 was obtained with Linear Discriminant Function Analysis.

For ANN, highest performance measures, in comparison, were obtained with following conditions- one input layer with 46 units, standardised rescaling of the sex predictor, and sigmoid activation, one hidden layer with two units, sigmoid activation function with 2 units within the middle layer, and a hyperbolic tangent activation function with 2 units for the output layer. Thus, accuracy percentages obtained when using these attributes, alone, have been reported herein.

Prior to undertaking SVC, the “matplotlib. pyplot” package within Python was utilised to generate a scatter plot for the training set data. Obtained plot indicated that a linear separation between data is possible and most apt. Thus, Support Vector Classification was further undertaken with a linear kernel, and accuracy values reported here correspond to those obtained when using a linear kernel.

In accordance with previous investigations, which indicated no significant differences in accuracy between different growing methods [27], a Decision tree was constructed using the CRT (classification and regression) growing method alone. Accuracy values reported here correspond to CRT.

kNN classification was undertaken with different k values- 5, 10, 25, 50, 75, and 100. Amongst these, k values of 50 and 75 garnered highest accuracy percentages, and thus, only these have been reported here.

### Accuracy for sex estimation

Accuracy percentages obtained with different sex estimation models are listed in Table 3. By and large, higher accuracy percentages were obtained for the training set with different statistical modalities utilised herein. The only exceptions to this finding were accuracy percentages obtained with QDFA in males, and prediction values computed using neural networks in females. Overall, statistically significant differences were observed between the accuracy percentages computed for the training and test sets with different models (*p* < 0.05). Between the two biological sexes, higher prediction percentages were observed in females using QDFA for the training set, LRA for the test set, neural networks for the test set, SVC for the test set, DTC for the training and test sets, and kNN for the test set. For all other computations, males garnered higher accuracy percentages.

**Table 3:** Accuracy for sexing with different statistical approaches, grouped by training and test cohorts

Statistical approach	Study set	Sex	Accuracy (%)
LDFA	Training set (*N* = 567)	Female	79.34%
		Male	86.25%
		Total population	82.89%
	Test set (*N* = 101)	Female	77.77%
		Male	80.35%
		Total population	79.20%
QDFA	Training set (*N* = 567)	Female	81.50%
		Male	81.35%
		Total population	81.30%
	Test set (*N* = 101)	Female	75.00%
		Male	83.01%
		Total population	79.20%
LRA	Training set (*N* = 567)	Female	80.07%
		Male	82.81%
		Total population	81.30%
	Test set (*N* = 101)	Female	80.00%
		Male	78.57%
		Total population	79.20%
ANN	Training set (*N* = 567)	Female	79.10%
		Male	87.70%
		Total population	83.50%
	Test set (*N* = 101)	Female	83.70%
		Male	76.70%
		Total population	79.60%
SVC	Training set (*N* = 567)	Female	80.07%
		Male	82.47%
		Total population	81.30%
	Test set (*N* = 101)	Female	80.00%
		Male	78.57%
		Total population	79.20%
DTC	Training set (*N* = 567)	Female	89.85%
		Male	77.66%
		Total population	83.59%
	Test set (*N* = 101)	Female	86.66%
		Male	71.42%
		Total population	78.21%
kNN	Training set (*N* = 567)	Female	80.07%
		Male	82.47%
		Total population	81.30%
	Test set (*N* = 101)	Female	80.00%
		Male	78.57%
		Total population	79.20%

LDFA = Linear Discriminant Function Analysis; QDFA = Quadratic Discriminant Function Analysis; LRA = Logistic Regression Analysis; ANN = Artificial Neural Network; SVC = Support Vector Classification; DTC = Decision Tree Classification; kNN = k Nearest Neighbour

Highest accuracy values were achieved using DTC in the pooled sample and in females and ANN in males, all of them in training sets, meanwhile lowest ones were found with LDFA, QDFA, LRA, SVC and kNN in the total population, QDFA in females and DTC in males, all of them in test sets. However, accuracy values garnered for the training set indicated no statistically significant differences when using different statistical approaches. The same finding was re-enforced, with no statistically significant differences observed between computed accuracy values for the test set when using different statistical approaches. Similarly, no statistically significant differences were observed between accuracy percentages computed for males and females of the training set, as well as the test set, using different statistical models.

### ROC curves and AUC values computed for the test set

**Fig. 2:**
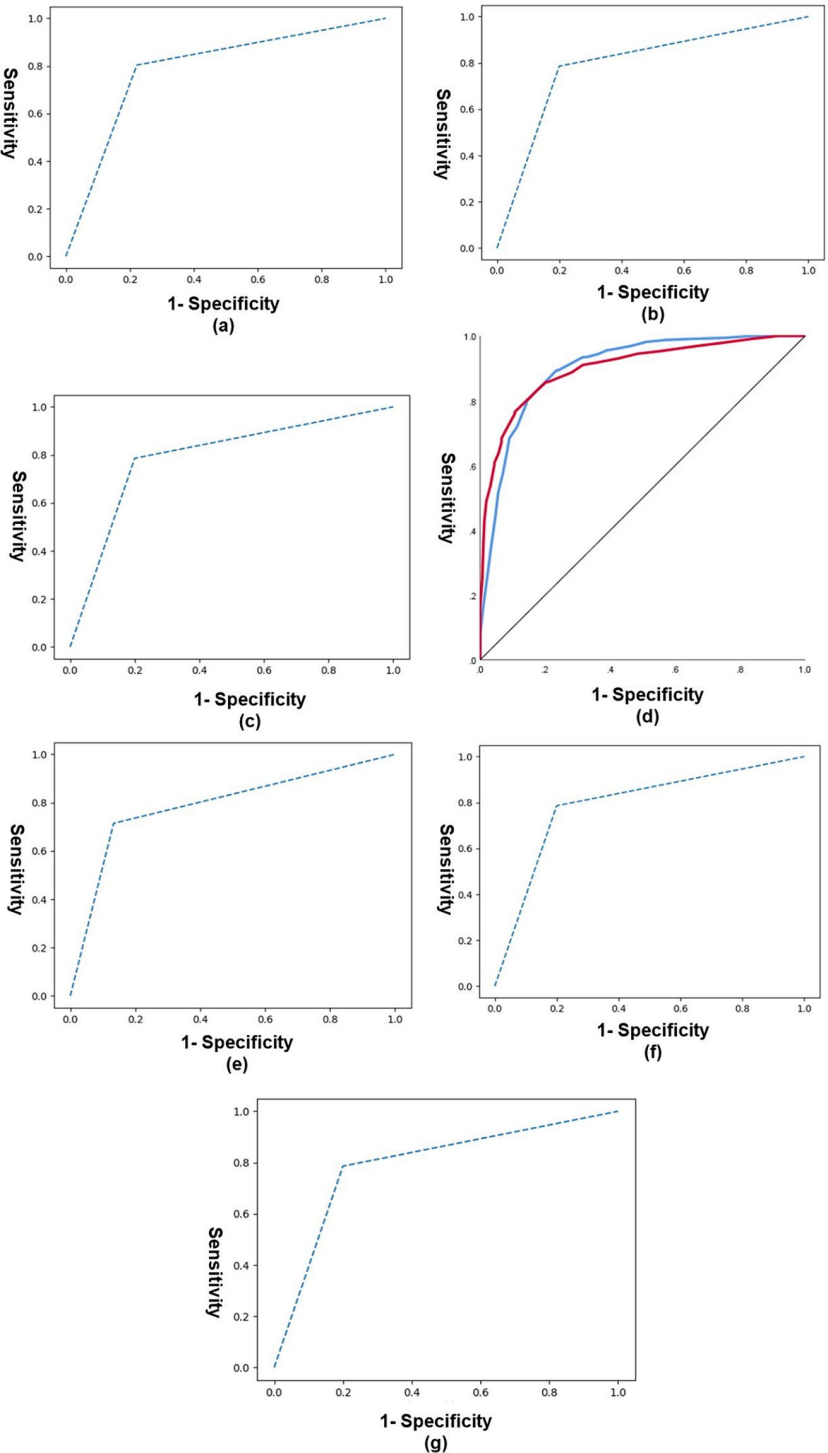
ROC curves obtained with different statistical approaches for the test set (*N* = 101). (**a**) LDFA; (**b**) QDFA; (**c**) LRA; (**d**) ANN; (**e**) SVC; (**f**) DTC; (**g**) kNN. Blue line indicates females and red line indicates males in (**d**)

Area under the curve (AUC) values associated with ROC curves were estimated with different statistical approaches. AUC curves offer a comprehensive measure of model performance (here binary classification performance), with a greater AUC value denoting a higher discriminatory power [71]. AUC values associated with ROC curves plotted for the test set using different statistical modalities are shown in Table 4. Highest AUC value was obtained with neural networks, and lowest with Linear discriminant analysis. Corresponding ROC curves for all models are plotted in Fig. 2.

**Table 4:** AUC values obtained with different statistical modalities for the test set (*N* = 101)

Statistical approach	AUC value
LDFA	0.790
QDFA	0.792
LRA	0.792
ANN	0.901
SVC	0.792
DTC	0.792
kNN	0.792

LDFA = Linear Discriminant Function Analysis; QDFA = Quadratic Discriminant Function Analysis; LRA = Logistic Regression Analysis; ANN = Artificial Neural Network; SVC = Support Vector Classification; DTC = Decision Tree Classification; kNN = k Nearest Neighbour; AUC = Area under curve

## Discussion

The estimation of biological sex is a prerequisite for human identification, particularly within the contexts of archaeological, medico-legal, and forensic investigations, mandating professionals to be trained in applying updated and robust approaches for identification. Different bony elements across the skeletal framework have proven to be reliable markers of sex, as reported within literature, although classical methodologies remain focused on the skull and pelvic anatomical differences.

The pelvis, in addition to its role in parturition, is credited with having a higher survivability when compared to other elements within the skeletal framework. This, in conjunction with its established applicability for human identification renders it an extremely reliable skeletal marker within diverse contexts. Within the human pelvis, the acetabulum, as an individual element, is known to provide meaningful information regarding age-at-death [72–76], biological sex [2, 4, 12, 26], and even stature [77]. Furthermore, in agreement with the present results, previous research has indicated a good reliability during inter and intra-observer error estimations using different acetabular variables [4, 12, 73, 78], rendering the acetabulum a complete and excellent anatomical evidence. In specific contexts, like past population studies or special forensic ones (e.g. terrorist attacks, natural disasters, big fires, train or plain accidents…), where the preservation of samples is scarce, the acetabulum, may thus prove to be vital for profiling, more so in cases where few skeletal markers are viable for examination. However, specific research on sex estimation using the acetabulum is currently limited in Iberian biogeographical contexts.

Statistical testing using the Mann-Whitney U test revealed a significant sexual dimorphism in the acetabular diameter. Such a finding is to be expected, given the differences in robusticity and the role of the pelvis, and by extension, the acetabulum, in childbearing [19]. Our findings pertaining to sexual dimorphism of the acetabulum are corroborated by previous investigations [2, 4, 14, 19, 23–25, 44]. Acetabular diameter values were observed to be higher in case of males for all three population groups in the present study, which is in agreement with previous studies [4, 12, 14, 16, 18–20, 22–25, 44, 56, 79–81]. This can be attributed to the greater stress imposed by muscular development and weight bearing in males [16, 82].

## Different mathematical approaches for sex estimation

### Traditional approaches

Discriminant Function Analysis is a commonly preferred method for sex estimation as it renders prediction and subsequent identification more mathematical, objective, and direct [17]. However, DFA has several assumptions which must be, under ideal scenarios, satisfied prior to its application. Discriminant analysis, being a parametric approach, assumes a normal distribution and homogeneity of the variance-covariance matrix. The method is sensitive to outliers, and in order to avoid overfitting requires a large enough sample size, i.e., at least 3–4 times the number of independent variables [27, 62, 63]. DFA has previously been employed for sex estimation with different markers across the skeletal framework [83–89], including the acetabulum [2, 4, 12, 16–18, 20, 24, 25, 44, 79]. Such previous investigations highlighted the vertical acetabular diameter as one amongst the most discriminatory sex variables [17, 19, 25, 44, 81]. Discriminant Function Analysis in the present study indicates that sex can be differentiated through a demarking point, with males on the higher side (positive group centroid value) and females on the lower side (negative pole). Patriquin et al. [19], Bubalo et al. [4], Steyn & Iscan [23], and Patriquin [44] reported similar findings for South African, Croatian, Greek, and African populations, respectively. Accuracy percentages obtained with LDFA across numerous studies are listed in Table 5. Accuracy obtained with males of the training set in the present study are comparable to those reported previously [2, 4, 12, 18, 19, 22, 23] while accuracy obtained with males of the training and test set in the present study are higher than those reported by Patriquin for South African white males [19], and Steyn and Patriquin for South African and Greek populations [24]. Cross-validation accuracies reported by Macaluso for a French male population [78] are higher than those obtained here for an Iberian male test population. For females of the training set, however, accuracy percentages obtained herein were higher than those reported by Patriquin for black females [19], alone. With females of the test set, lowest accuracy percentages were obtained with the present Iberian population when compared to previous studies [19, 78]. For the total population, with the training set of the Iberian population, lowest accuracy percentages were obtained here with the exception of Steyn and Patriquin [24] who reported a marginally lower accuracy of 82.50%. For the test set/ cross-validation, the present study reported lowest accuracy percentages. These differences in accuracy percentages, however, were not statistically significant. It is also prudent to mention here the lack of homogeneity with regards to test groups and cross-validation. While the present study employed a test group or holdout group of 15% of the total study sample, certain other investigations utilised a LOOCV (leave-one-out cross validation). This, too, could have contributed, in part, to these observed differences, albeit non-significant, in accuracy.

**Table 5:** Sex estimation accuracy with the acetabular diameter, reported across literature

			Male		Female		Total population	
Study	Population affinity	Statistical approach	Training set	Test set/ CV	Training set	Test set/ CV	Training set	Test set/ CV
Murphy [18]	Polynesian	LDFA	-	-	-	-	85.20–86.20%	-
Patriquin et al. [19]	South African	LDFA	77.00–89.00%	77.00–89.00%	78.00–86.00%	78.00–86.00%	-	-
Benazzi et al. [2]	Italian	LDFA	-	-	-	-	95.20%	-
Steyn & Iscan [23]	Greek	LDFA	87.00%	87.00%	80.90%	80.90%	83.90%	83.90%
Papaloucas et al. [22]	Greek	LDFA	-	-	-	-	87.00%	87.00%
Steyn & Patriquin [24]	South African & Greek	LDFA	80.50%	80.50%	84.40%	84.40%	82.50%	82.50%
Macaluso [12]	French	LDFA	82.60–87.00%	-	80.60–88.90%	-	84.10–85.40%	-
Gómez-Valdés et al. [25]*	Mexican	LDFA	-	-	-	-	85.30–86.80%	-
Bubalo et al. [4]	Croatian	LDFA	84.00	-	90.00%	-	87.00%	-
Macaluso [77]**	French	LDFA	-	90.00%	-	81.50%	-	86.60%
Present study	Iberian	LDFA	86.25%	80.35%	79.34%	77.77%	82.89%	79.20%
Macaluso [77]**	French	LRA	95.00	-	81.50%	-	89.60%	-
Present study	Iberian	LRA	82.81%	78.57%	80.07%	80.00%	81.30%	79.20%

LDFA = Linear Discriminant Function analysis; LRA = Logistic Regression Analysis; CV = Cross-validation

*Reported values for multivariable analysis, one parameter out of which was the acetabular diameter

**Values have been reported only for left acetabulum to maintain continuity with the present study

In comparison to LDFA, QDFA yielded marginally higher accuracy percentages for females of the training set. Males and the total population, however, garnered lower accuracy with QDFA. With males of the test set, QDFA gave higher accuracy percentages, whereas for females and the total population, LDFA yielded higher accuracy. Such varying patterns in accuracy call into question intervening factors which can influence the association between acetabular diameter and sex. Future investigations should attempt to establish the influence of such factors, for example age, stature or body mass index, on the accuracy of the acetabular diameter to estimate sex. It is highly plausible that when such additional factors are taken into consideration, QDFA might present as the more accurate statistical approach, attributable to its use of a more flexible i.e., quadratic decision boundary. It is also important to note here that all assumptions associated with DFA were not satisfied in the present study, primarily with regards to normality of the sample. This, too, could have resulted in the inconsistencies in accuracy percentages observed with the linear and quadratic approach.

Logistic Regression Analysis constitutes another commonly utilised statistical approach for sex estimation [38, 41, 42, 90–93]. LRA, being a semiparametric approach has fewer assumptions to satisfy when compared to DFA, i.e., primarily a large sample size is warranted. LRA is more flexible in comparison to DFA and does not mandate a normally distributed sample, linearly related predictor variables, homoscedasticity, and works well with both discrete, as well as continuous data [27]. Despite this inherent flexibility, the use of LRA for sexing the acetabulum is relatively unreported [12, 75, 80]. Furthermore, Nagesh et al. [80] employed the acetabulum-pubis index for sex estimation, as opposed to just the acetabular diameter, and measured the acetabular diameter using a procedure different from the present study. Macaluso [78], on the other hand, employed the acetabular diameter and Logistic Regression Analysis for sex estimation and reported accuracies ranging from 84.10 to 89.60%, higher than those obtained herein with Iberian populations (Table 5).

It is, however, worth mentioning that discrepancies in accuracies between aforementioned research studies, and the present study, could also be attributed to the underlying sexual dimorphism present in the population (s) under scrutiny. Possible sex differences between the populations studied in previous researches, in comparison to the present study sample, may be one of the factors responsible for the observed differences.

ROC curve plotted with the aforementioned traditional statistical methods demonstrated an acceptable discrimination power [71] between sexes when using this acetabular attribute.

### Machine learning approaches

Artificial Neural Networks, a form of supervised Machine Learning, is being increasingly incorporated into sex estimation investigations [94–97]. Unlike DFA, ML, and by extension ANN, does not mandate satisfying any assumptions regarding distribution of the sample. An extensive literature search revealed that ANN has not been employed for sexing the acetabulum so far. As a result, findings of our study could not be corroborated by previous literature. However, convolutional neural networks have previously been utilised to estimate sex from the acetabular morphology and yielded an accuracy of 74.60% [98]. The neural networks used within the present research were built using different activation functions- hyperbolic tangent and sigmoid within the input and hidden layers, and hyperbolic tangent, sigmoid, and softmax within the output layer. Given that the required output is in the form of 0,1, sigmoid activation presents as the ideal choice for input and hidden layers, whereas softmax activation is apt for the output layer as the intended objective is to undertake classification of subjects into mutually exclusive classes. However, hyperbolic tangent within the output layer yielded higher accuracy, when compared to the softmax function. Greater in-depth research into how varying activation functions can impact observed sex estimation accuracy, and possible reasons for this, is currently wanting.

Support Vector Machines, another class of supervised Machine Learning approaches enable both, regression, as well as classification. An effective application of SVC warrants that the data be linearly separable and this is often achieved through the use of kernels. An advantage of SVC for sexing is that it is applicable even with smaller datasets. While SVC has previously been utilised for sex estimation with different skeletal markers [46, 99, 100], its usage for sexing the acetabulum is presently lacking. This prevented a comparative evaluation of our results. Preliminary scatter plot evaluations of the training set data points indicated that a linear separation is feasible for the data at hand i.e., males and females occupy, by and large, different spaces which can be linearly separated. This finding is corroborated by both, centroid values obtained with DFA [4, 19, 23, 44], as well as the known anatomical size differences between the two sexes [26]. Linear kernels are additionally advantageous as they are often simpler and quicker to train. Nevertheless, given the occasionally higher accuracy observed with QDFA, it might be beneficial to incorporate additional factors such as age, and investigate sexing accuracy using polynomial kernels within future investigations. In fact, the dynamic shape metamorphosis of the pelvis across adult human lifespan has been already noticed [101].

Decision Trees are another mode of non-parametric supervised Machine Learning which allow for regression and classification problems. Being, primarily, a non-statistical approach Decision Trees require no assumptions regarding distribution, or variance. However, they do warrant certain non-statistical assumptions such as the discretization of continuous variables. Decision Trees have previously been utilised for sexing the pelvis [27, 63], and also the acetabulum [98, 102]. However, Yusuf et al. [102] reported cumulative accuracies (involving multiple variables pooled together) for sexing with Decision Trees, whereas, Cao et al. [98] focussed their investigation towards morphological sex differences of the acetabulum. Different Decision Trees can be constructed using specific growing methods such as CHAID (Chi-square Automatic Interaction Detection), CRT (Classification and Regression Trees), QUEST (Quick, Unbiased, Efficient Statistical Tool), etc. CHAID and CRT trees carry out splits using a chi-squared test and computations of Gini impurity, respectively. QUEST trees, on the other hand, split on the assumption that the target variable is a continuous variable. The three growing methods differ not only on the splitting method employed, but also the kind of data they can handle. While CHAID and QUEST work with categorical variables, CRT works equally well with categorical and continuous data. Klales et al. utilised all three growing methods for sexing the pelvis and reported similar accuracy values using all three [27]. In keeping with these findings, and with the data flexibility accorded by CRT, this method alone was employed within the present study. Accuracy obtained with DTC herein could not be compared with previous literature due to lack of similar data. Future investigations should attempt to decipher how the use of different growing algorithms can impact accuracy and bias associated with sexing the acetabulum, if at all.

kNN is a type of non-parametric supervised ML classification approach, and thus does not warrant satisfying any assumptions regarding sample distribution. Classification within kNN ensues on the basis of patterns observed within the data, as opposed to predetermined labels. k in kNN denotes the most similar individuals in a reference sample (training set), and subsequent classification is undertaken based on group identities of these similar individuals [27]. kNN algorithms have previously been utilised for sexing the pelvis [27, 49]. However, there is a scarcity of literature regarding its application for sexing the acetabulum. Since kNN classification relies greatly on the nearest neighbour, different k values can impact obtained accuracy values significantly. Within the present study, k of 50 and 75 proved to be most reliable for sex estimation. Further attempts should be made to utilise different ‘k’ in order to better understand the performance of kNN classification as a result of varying k values.

ROC curve plotted using machine learning approaches garnered an excellent discrimination [71] with ANN, and acceptable discrimination using DFA, LRA, SVC, DTC, and aforementioned activation functions yielded an outstanding discrimination [71] between the two sexes.

In the present study, DTC yielded highest accuracy percentages for females and the combined population, and ANN garnered the most accurate results for males. The improved performance observed with ML approaches herein, is in agreement with previously undertaken studies [45, 46, 100, 103–107]. High performance measures observed with DTC can be attributed to the inherent pruning characteristic of Decision Trees which prevents overfitting of data. Furthermore, the use of a single variable in the present study could have also contributed to the observed high accuracy through the creation of a simple tree with pure leaf nodes, as opposed to complex trees with consistently declining purity. Artificial Neural Networks, in turn, generate high accuracy by modelling heteroscedasticity much more efficiently, as well as its ability to predict the unseen/ unknown through generalization. A high AUC value for ANN ROC curves further validates the accurate performance of Neural Networks (Table 4).

Males of the training set garnered higher accuracy percentages in comparison to females with most statistical approaches, with the exception of QDFA and DTC. For the test set, however, by and large, higher accuracy percentages were observed in females. The only exceptions to this dictum were LDFA and QDFA, wherein males of the test set demonstrated higher accuracy. Previously undertaken investigations with LDFA and LRA have also indicated such variable findings, with certain studies reporting higher accuracy in males [12, 19, 23, 44, 78], and certain others illustrating higher accuracy percentages for females [4, 12, 19, 24, 44]. It has previously been hypothesized that opposing pressures of locomotion and obstetrics result in lesser variability within the female pelvis [108]. Hence, in theory, greater accuracies should be observed with the gracile sex. However, the findings of the present study contradict this hypothesis, agreeing with the theory put forth by Kurki [109]. A plausible explanation for lower female accuracy, observed across studies, could be the effects of decreased hormonal expression with increasing age. As previously highlighted, future investigations with the acetabular diameter will attempt to examine the effects of age on sexing accuracy. Such investigations could help address queries raised within the present research, primarily the variable accuracy patterns observed between sexes. Varying patterns of accuracy percentages observed within the present study and other similar investigations could also be attributed to the unequal representation of the two sexes. Similar investigations should be undertaken, ensuring an equal representation of both biological sexes, in order to corroborate/ validate these findings.

### Applicability and future lines of research

The highest accuracy obtained with the acetabular diameter for sex-pooled Iberian populations using different statistical approaches, across both training and test cohorts, was 83.59%. This is comparable to the values reported previously [12, 18, 23–25]. However, higher accuracy percentages have been reported for Italian [2], Greek [22], and Croatian [4] populations. Highest accuracy obtained with Iberian females was 89.85%, comparable to those reported for French [12] and Croatian [4] populations, and higher than other investigations [19, 23, 24, 44]. Highest accuracy percentage obtained with Iberian males was 87.70%, which is comparable to the values reported previously [4, 12, 19, 23, 24, 44]. Given the durability and taphonomic resilience associated with the acetabulum, along with the obtained accuracy values, this skeletal element presents as an efficient sex/ human identification marker, more so in scenarios where other more accurate markers are rendered non-viable [110].

Nevertheless, in order to promote and employ such reliable, alternate markers for sex estimation, and by extension, human identification, standard methodological and statistical approaches need to be incorporated. Regarding the present variable, the acetabular diameter has been defined and measured slightly differently across the scientific literature (Table 5). While, Djorojevic et al. [35] performed the Genovés’ approach [37] (*vertical acetabular diameter*,* maximum*,* taken perpendicularly to the symphysis pubis width*,* following the general axis of the ischium body*,* or perpendicular to the ascendant pubic ramus*), Macaluso [12, 78] followed Murphy’s approach [18] (*from the point on the superior margin of the acetabulum where the rim intersects the anterior border of the ilium to the most distant point on the inferior margin of the acetabulum*). Benazzi et al. [2] defined the acetabular diameter as the *projection of the straight line passing through the anterior horn of the acetabular rim and the centre of the acetabular depression*, Papaloucas et al. [22] simply defined it *between its rims (anterior-posterior)*, without any original reference, and Murail et al. [9] used the definition of Bräuer [36] (*maximum vertical diameter of the acetabulum*,* on the acetabular rim*,* as a prolongation of the longitudinal axis of the ischium)*, the most similar to the one used here [56]. Finally, Steyn and Iscan [23], Steyn and Patriquin [24], Gómez-Valdés et al. [25], Patriquin et al. [19] or Bubalo et al., [4] followed the description of Kelley [111], with slight modifications to the original description. While Patriquin et al. [19] described it *from the middle of the ridge on the superior border of the acetabulum to the inferior border*, Steyn and Patriquin [24] and Steyn and Iscan [23] defined the maximum diameter of the acetabulum measured in a *superior to inferior direction;* Bubalo et al. [4] or Gómez-Valdés et al. [25], on the other hand, added *along the axis of the body of the ischium* to the previous definition. However, despite the cited previous differences between definitions and its potential controversy or impossibility to make comparisons, any acetabular diameter definition has been proved to achieve good levels of sexing reliability. Further research should examine if actual significant differences would exist between the diverse acetabular diameter definitions/measurements.

In addition to this potentially needed methodological standardization, our study also advocates moving towards Machine Learning targeted approaches for data analysis. While accuracy and performance measures obtained with different ML approaches are largely comparable to those reported by traditional statistical practices, ML circumvents the issue of satisfying numerous assumptions, which are, more often than not, flouted during sex estimation-based research. Furthermore, while the Measurement Statistics Controversy [27, 112] argues that the focus should be on obtaining more accurate results, as opposed to abiding by numerous statistical assumptions, the use of ML can help do away with this issue and confusion in entirety. The present study employed the use of three different classes of statistics- traditional (DFA, LRA), lazy-learning (kNN), and ML (ANN, DTC, SVC) to assess this impact of illegal statisticizing. Between these approaches of traditional statistics wherein assumptions were not satisfied, lazy-learning approaches where no characteristic training was undertaken, and ML techniques which required no specific assumptions to be fulfilled, ML yielded the best results. Given the absence of any significant population differences in sexing with the acetabular diameter [24], creating an ML application, which has been trained using data collected from around the globe, can help simplify the process of sex estimation for experts, as well as novice practitioners. Sex estimation using the acetabulum can be rendered even more accurate by permitting automatic detection of landmarks using Artificial Intelligence, evading the subjectivity associated with identifying landmarks.

The present study indicated that through the use of one of the standardised anatomical definitions for the acetabular diameter and ML, the existing accuracy and applicability of this parameter as a univariable sex marker can be further augmented. Nevertheless, the utility of multivariable sexing approaches cannot be overlooked. Future investigations will be targeted at incorporating a multivariable approach, including and not limited to additional acetabular parameters, and comparing multivariable sexing accuracy against individual univariable approaches. A present limitation associated with this study is the non-estimation of inter-observer repeatability. Regardless, multiple investigations with Bräuer’s description of the acetabular diameter [36] (similar to the definition utilised here) have indicated good repeatability within and between observers [8, 29, 33, 34, 60, 61, 113], demonstrating the utility of this marker for human identification.

## Conclusion

The proven sexual dimorphism of the acetabulum along with its higher preservation within diverse and complex skeletal contexts renders this anatomical element vital for human identification. Findings of the present research indicate that Machine Learning approaches garner better accuracy outcomes for sex estimation, specifically DTC in the sex-pooled sample and females, and ANN in males. As an added advantage, the assumptions that need to be fulfilled to utilize Machine Learning modalities for human identification are rather limited and unrestrictive. A combination of these advantages renders ML a field to strongly consider within future investigations. In order to maximize the applicability of this statistical modality and make its use easier within field contexts, implementing a freely available software where any practitioner, bioarcheologist or anthropologist could enter their data to quickly ascertain biological sex, along with the associated likelihood, should be the next stage.

## Data Availability

N/A.

## References

[1] Iscan MY, Steyn M (2013) The human skeleton in forensic medicine. Charles C Thomas

[2] Benazzi S, Maestri C, Parisini S, Vecchi F, Gruppioni G (2008) Sex assessment from the acetabular rim by means of image analysis. Forensic Sci Int 180. 10.1016/j.forsciint.2008.06.007. :58.e1-58.e3 10.1016/j.forsciint.2008.06.007 18692971

[3] Mahakkanukrauh P, Ruengdit S, Tun SM, Case DT, Sinthubua A (2017) Osteometric sex estimation from the os coxa in a Thai population. Forensic Sci Int 271. 10.1016/j.forsciint.2016.11.043. :127.e1-127.e7 10.1016/j.forsciint.2016.11.043 28062152

[4] Bubalo P, Baković M, Tkalčić M, Petrovečki V, Maye D (2019) Acetabular osteometric standards for sex estimation in contemporary Croatian population. Croat Med J 60:221–226. 10.3325/cmj.2019.60.221 31187949 10.3325/cmj.2019.60.221 PMC6563177

[5] Gillet C, Costa-Mendes L, Rérolle C, Telmon N, Maret D, Savall F (2020) Sex estimation in the cranium and mandible: a multislice computed tomography (MSCT) study using anthropometric and geometric morphometry methods. Int J Legal Med 134:823–832. 10.1007/s00414-019-02203-0 31897666 10.1007/s00414-019-02203-0

[6] Moore MK, DiGangi EA, Niño Ruíz FP, Davila OJH, Medina CS (2016) Metric sex estimation from the postcranial skeleton for the Colombian population. Forensic Sci Int 262:286e1–286e8. 10.1016/j.forsciint.2016.02.018 10.1016/j.forsciint.2016.02.018 27032896

[7] Fasemore T, Bidmos M, Mokoena P, Imam A, Billings BK, Mazengenya P (2018) Dimensions around the nutrient foramina of the tibia and fibula in the estimation of sex. Forensic Sci Int 287:222e1. 10.1016/j.forsciint.2018.03.015 10.1016/j.forsciint.2018.03.015 29678345

[8] Brůžek J, Santos F, Dutailly B, Murail P, Cunha E (2017) Validation and reliability of the sex estimation of the human os coxae using freely available DSP2 software for bioarchaeology and forensic anthropology. Am J Phys Anthropol 164:440–449. 10.1002/ajpa.23282 28714560 10.1002/ajpa.23282

[9] Murail P, Brůžek J, Houët F, Cunha E (2005) DSP: a tool for probabilistic sex diagnosis using worldwide variability in hip-bone measurements. Bulletins et mémoires de la Société d’Anthropologie de Paris BMSAP 17:167–176. 10.4000/bmsap.1157

[10] Coleman WH (1969) Sex differences in the growth of the human bony pelvis. Am J Phys Anthropol 31:125–151. 10.1002/ajpa.1330310202 5348790 10.1002/ajpa.1330310202

[11] Loth SR, İşcan MY (2000) Anthropology. Morphological Age Estimation. In: Encyclopedia of Forensic Sciences. pp 242–252

[12] Macaluso PJ (2010) Sex determination from the acetabulum: test of a possible non-population-specific discriminant function equation. J Forensic Leg Med 17:348–351. 10.1016/j.jflm.2010.04.011 20650427 10.1016/j.jflm.2010.04.011

[13] Sorg MH, Haglund WD (1996) Forensic taphonomy: the postmortem fate of human remains. CRC 10.1136/bmj.319.7207.458 PMC1127062 10445946

[14] Davivongs V (1963) The pelvic girdle of the Australian aborigine; sex differences and sex determination. Am J Phys Anthropol 21:443–455. 10.1002/ajpa.1330210403 14185525 10.1002/ajpa.1330210403

[15] Seidler H (1980) Sex-diagnosis of isolated Os coxae by discriminant functions. J Hum Evol 9:597–600. 10.1016/0047-2484(80)90088-3

[16] Dibennardo R, Taylor JV (1983) Multiple discriminant function analysis of sex and race in the postcranial skeleton. Am J Phys Anthropol 61:305–314. 10.1002/ajpa.1330610305 6614145 10.1002/ajpa.1330610305

[17] Rissech C, Malgosa A (1997) Sex prediction by discriminant function with central portion measures of innominate bones. Homo 48:22–32

[18] Murphy AMC (2000) The acetabulum: sex assessment of prehistoric New Zealand Polynesian innominates. Forensic Sci Int 108:39–43. 10.1016/S0379-0738(99)00206-6 10697777 10.1016/s0379-0738(99)00206-6

[19] Patriquin ML, Steyn M, Loth SR (2005) Metric analysis of sex differences in South African black and white pelves. Forensic Sci Int 147:119–127. 10.1016/j.forsciint.2004.09.074 15567615 10.1016/j.forsciint.2004.09.074

[20] Dixit SG, Kakar S, Agarwal S, Choudhry R (2007) Sexing of human hip bones of Indian origin by discriminant function analysis. J Forensic Leg Med 14:429–435. 10.1016/j.jflm.2007.03.009 17720595 10.1016/j.jflm.2007.03.009

[21] Rösing FW, Graw M, Marré B et al (2007) Recommendations for the forensic diagnosis of sex and age from skeletons. Homo 58:75–89. 10.1016/j.jchb.2005.07.002 17306261 10.1016/j.jchb.2005.07.002

[22] Papaloucas C, Fiska A, Demetriou T (2008) Sexual dimorphism of the hip joint in greeks. Forensic Sci Int 179. 10.1016/j.forsciint.2008.03.007. :83.e1–3 10.1016/j.forsciint.2008.03.007 18455335

[23] Steyn M, İşcan MY (2008) Metric sex determination from the pelvis in modern greeks. Forensic Sci Int 179:86. e1-86.e6 10.1016/j.forsciint.2008.04.022 18554832

[24] Steyn M, Patriquin ML (2009) Osteometric sex determination from the pelvis—does population specificity matter? Forensic Sci Int 191. 10.1016/j.forsciint.2009.07.009. :113.e1-113.e5 10.1016/j.forsciint.2009.07.009 19665855

[25] Gómez-Valdés JA, Torres Ramírez G, Báez Molgado S, Sain-Leu PH, Caballero JLC, Sánchez‐Mejorada G (2011) Discriminant function analysis for sex assessment in pelvic girdle bones: sample from the contemporary Mexican population. J Forensic Sci 56:297–301. 10.1111/j.1556-4029.2010.01663.x 21265837 10.1111/j.1556-4029.2010.01663.x

[26] San-Millán M, Rissech C, Turbón D (2017) Shape variability of the adult human acetabulum and acetabular fossa related to sex and age by geometric morphometrics. Implications for adult age estimation. Forensic Sci Int 272:50–63. 10.1016/j.forsciint.2017.01.005 28113134 10.1016/j.forsciint.2017.01.005

[27] Klales AR, Ousley SD, Passalacqua NV (2020) Statistical approaches to sex estimation. In: Klales AR (ed) Sex estimation of the human skeleton. Academic Press, pp 203–217

[28] Mestekova S, Bruzek J, Veleminska J, Chaumoitre K (2015) A test of the dsp sexing method on ct images from a modern French sample. J Forensic Sci 60:1295–1299. 10.1111/1556-4029.12817 26258990 10.1111/1556-4029.12817

[29] de Almeida SM, de Carvalho MVD, de Lyra Menezes MCT, Petraki GGP, Cunha E, Soriano EP (2020) Validation of the DSP2 tool in a contemporary identified skeletal collection from northeastern Brazil. Adv Anthropol 10:169–180. 10.4236/aa.2020.102010

[30] Sánchez-Mejorada G, Gómez-Valdés J, Herrera P, Veleminsky P, Bruzek J (2011) Valoración Del método de Diagnóstico sexual Probabilístico (DSP) en una colección osteológica mexicana. Estudios De Antropología Biológica 15. 10.22201/iia.14055066p.2011.42780

[31] Quatrehomme G, Radoman I, Nogueira L, du Jardin P, Alunni V (2017) Sex determination using the DSP (probabilistic sex diagnosis) method on the coxal bone: efficiency of method according to number of available variables. Forensic Sci Int 272:190–193. 10.1016/j.forsciint.2016.10.020 27856048 10.1016/j.forsciint.2016.10.020

[32] Rajasekhar S (2017) Sex determination by biometry of anterior features of human hip bones in South Indian population. J Clin Diagn Res. 10.7860/JCDR/2017/27927.10051 28764142 10.7860/JCDR/2017/27927.10051 PMC5535335

[33] Machado MPS, Costa ST, Freire AR et al (2018) Application and validation of Diagnose Sexuelle Probabiliste V2 tool in a miscegenated population. Forensic Sci Int 290:351–e1. 10.1016/j.forsciint.2018.06.043 10.1016/j.forsciint.2018.06.043 30077496

[34] Kranioti EF, Šťovíčková L, Karell MA, Brůžek J (2019) Sex estimation of os coxae using DSP2 software: a validation study of a Greek sample. Forensic Sci Int 297. 10.1016/j.forsciint.2019.02.011. :371.e1-371.e6 10.1016/j.forsciint.2019.02.011 30851999

[35] Djorojevic M, Roldán C, García-Parra P, Alemán I, Botella M (2014) Morphometric sex estimation from 3D computed tomography os coxae model and its validation in skeletal remains. Int J Legal Med 128:879–888. 10.1007/s00414-014-1033-x 24928326 10.1007/s00414-014-1033-x

[36] Bräuer G (1988) Osteometrie. In: Knussmann R (ed) Anthropologie, handbuch des vergleichenden biologie des menschen. Fischer, Stuttgart

[37] Genovés S (1959) Diferencias sexuales en El Hueso Coxal. Universidad Nacional Autónoma de México, Instituto de Historia, Dirección General de Publicaciones, México

[38] Toneva D, Nikolova S, Harizanov S et al (2018) Sex estimation by size and shape of foramen magnum based on CT imaging. Leg Med 35:50–60. 10.1016/j.legalmed.2018.09.009 10.1016/j.legalmed.2018.09.009 30268691

[39] Peleg S, Pelleg Kallevag R, Dar G, Steinberg N, Masharawi Y, May H (2020) New methods for sex estimation using sternum and rib morphology. Int J Legal Med 134:1519–1530. 10.1007/s00414-020-02266-4 32072241 10.1007/s00414-020-02266-4

[40] Ahmed AA, Koko AO, Bahar ME (2021) Estimation of sex based on the sterna of Sudanese adults using multidetector computed tomography: a comparison of discriminant function analysis and binary logistic regression. Homo 72:41–51. 10.1127/homo/2021/1358 33585858 10.1127/homo/2021/1358

[41] Golpinar M, Salim H, Ozturk S, Komut E, Sindel M (2022) Sex estimation with morphometric and morphological characteristics of the crista galli. Surg Radiol Anat 44:1007–1015. 10.1007/s00276-022-02971-2 35750936 10.1007/s00276-022-02971-2

[42] Rani D, Krishan K, Kanchan T (2023) A methodological comparison of discriminant function analysis and binary logistic regression for estimating sex in forensic research and case-work. Med Sci Law 63:227–236. 10.1177/00258024221136687 36366800 10.1177/00258024221136687

[43] Novotný V (1986) Sex determination of the pelvic bone: a systems approach. Anthropologie 24:197–206

[44] Patriquin ML (2001) A comparative analysis of differences in the pelves of South African blacks and whites. University of Pretoria (South Africa)

[45] Knecht S, Santos F, Ardagna Y, Alunni V, Adalian P, Nogueira L (2023) Sex estimation from long bones: a machine learning approach. Int J Legal Med 137:1887–1895. 10.1007/s00414-023-03072-4 37526736 10.1007/s00414-023-03072-4

[46] Toneva D, Nikolova S, Agre G, Zlatareva D, Hadjidekov V, Lazarov N (2021) Machine learning approaches for sex estimation using cranial measurements. Int J Legal Med 135:951–966. 10.1007/s00414-020-02460-4 33179173 10.1007/s00414-020-02460-4

[47] Navega D, Vicente R, Vieira DN, Ross AH, Cunha E (2015) Sex estimation from the tarsal bones in a Portuguese sample: a machine learning approach. Int J Legal Med 129:651–659. 10.1007/s00414-014-1070-5 25186617 10.1007/s00414-014-1070-5

[48] Toy S, Secgin Y, Oner Z, Turan MK, Oner S, Senol D (2022) A study on sex estimation by using machine learning algorithms with parameters obtained from computerized tomography images of the cranium. Sci Rep 12:4278. 10.1038/s41598-022-07415-w 35277536 10.1038/s41598-022-07415-w PMC8917237

[49] d’Oliveira Coelho J, Curate F (2019) CADOES: an interactive machine-learning approach for sex estimation with the pelvis. Forensic Sci Int 302:109873. 10.1016/j.forsciint.2019.109873 31382223 10.1016/j.forsciint.2019.109873

[50] Pastor JF, Verona JAG, de Paz FJ, Barbosa E (1995) The anatomical museum of Valladolid. Yamaguchi J Vet Med 22:53–60

[51] Villoria Rojas C, Mata Tutor P, Labajo González E et al (2024) The identified skeletal Collection of the School of Legal Medicine: a contemporary osteological collection housed in Universidad Complutense De Madrid, Spain. Int J Legal Med 138:555–560. 10.1007/s00414-023-03047-5 37382705 10.1007/s00414-023-03047-5 PMC10861381

[52] Cardoso HFV (2006) Brief communication: the collection of identified human skeletons housed at the Bocage Museum (National Museum of Natural History), Lisbon, Portugal. Am J Phys Anthropol 129:173–176. 10.1002/ajpa.20228 16323180 10.1002/ajpa.20228

[53] Rissech C, García M, Malgosa A (2003) Sex and age diagnosis by ischium morphometric analysis. Forensic Sci Int 135:188–196. 10.1016/s0379-0738(03)00215-9 12927396 10.1016/s0379-0738(03)00215-9

[54] Rissech C, Malgosa A (2005) Ilium growth study: applicability in sex and age diagnosis. Forensic Sci Int 147:165–174. 10.1016/j.forsciint.2004.08.007 15567622 10.1016/j.forsciint.2004.08.007

[55] Schaefer M, Black S, Scheuer L (2009) Juvenile osteology: a laboratory and field manual. Academic, London

[56] Taylor JV, DiBennardo R (1984) Discriminant function analysis of the central portion of the innominate. Am J Phys Anthropol 64:315–320. 10.1002/ajpa.1330640314 6476103 10.1002/ajpa.1330640314

[57] San-Millán M (2013) Asimetrías en el hueso coxal: implicaciones en los métodos de estimación de la edad y determinación del sexo. In: Másteres de la UAM (ed) Año Académico 2010–2011, UAM Ediciones, Madrid

[58] Wayne WD, Daniel WW (1998) Biostatistics: a foundation for analysis in the health sciences, 7th edn. Wiley

[59] Koo TK, Li MY (2016) A Guideline of selecting and reporting Intraclass correlation coefficients for Reliability Research. J Chiropr Med 15:155–163. 10.1016/j.jcm.2016.02.012 27330520 10.1016/j.jcm.2016.02.012 PMC4913118

[60] Chapman T, Lefevre P, Semal P, Moiseev F, Sholukha V, Louryan S, Rooze M, Van Sint Jan S (2014) Sex determination using the probabilistic sex diagnosis (DSP: diagnose Sexuelle Probabiliste) tool in a virtual environment. Forensic Sci Int 234:189e1–18189. .e8 10.1016/j.forsciint.2013.10.037 24290894

[61] Rodriguez Paz A, Banner J, Villa C (2018) Validity of the probabilistic sex diagnosis method (DSP) on 3D CT-scans from modern Danish population. La Revue De Médecine Légale 10(2):43–49. 10.1016/j.medleg.2018.08.002

[62] Tabachnick BG, Fidell LS, Ullman JB (2013) Using multivariate statistics, 6th edn. Pearson Education, Boston

[63] Nikita E, Nikitas P (2020) Sex estimation: a comparison of techniques based on binary logistic, probit and cumulative probit regression, linear and quadratic discriminant analysis, neural networks, and naïve Bayes classification using ordinal variables. Int J Legal Med 134:1213–1225. 10.1007/s00414-019-02148-4 31444553 10.1007/s00414-019-02148-4

[64] Johnson RA, Wichern DW (2002) Applied Multivariate Statistical Analysis

[65] Narsky I, Porter FC (2013) Statistical analysis techniques in particle physics: fits, density estimation and supervised learning. Wiley

[66] Haykin S (2009) Neural networks and learning machines, 3rd edn. Pearson Education, India

[67] Ripley BD (2007) Pattern recognition and neural networks. Cambridge University Press

[68] Fan F, Dong X, Wu X et al (2020) An evaluation of statistical models for age estimation and the assessment of the 18-year threshold using conventional pelvic radiographs. Forensic Sci Int 314:110350. 10.1016/j.forsciint.2020.110350 32650207 10.1016/j.forsciint.2020.110350

[69] Tong S, Koller D (2001) Support vector machine active learning with applications to text classification. J Mach Learn Res 2:45–66

[70] Zhang Y (2012) Support Vector Machine classification algorithm and its application. In: Liu C, Wang L, Yang A (eds) Information Computing and Applications. Springer Berlin Heidelberg, Berlin, Heidelberg, pp 179–186

[71] Mandrekar JN (2010) Receiver operating characteristic curve in diagnostic test assessment. J Thorac Oncol 5:1315–1316. 10.1097/JTO.0b013e3181ec173d 20736804 10.1097/JTO.0b013e3181ec173d

[72] Rissech C, Estabrook G, Cunha E, Malgosa A (2006) Using the acetabulum to estimate age at death of adult males. J Forensic Sci 51:213–229. 10.1111/j.1556-4029.2006.00060.x 16566753 10.1111/j.1556-4029.2006.00060.x

[73] San-Millán M, Rissech C, Turbón D (2017) New approach to age estimation of male and female adult skeletons based on the morphological characteristics of the acetabulum. Int J Legal Med 131:501–525. 10.1007/s00414-016-1406-4 27363827 10.1007/s00414-016-1406-4

[74] Winburn AP (2019) Validation of the acetabulum as a skeletal indicator of age at death in modern european-americans. J Forensic Sci 64:989–1003. 10.1111/1556-4029.13972 30537265 10.1111/1556-4029.13972

[75] Belghith M, Marchand E, Ben Khelil M, Rougé-Maillart C, Blum A, Martrille L (2021) Age estimation based on the acetabulum using global illumination rendering with computed tomography. Int J Legal Med 135:1923–1934. 10.1007/s00414-021-02539-6 33713164 10.1007/s00414-021-02539-6

[76] Warrier V, Shedge R, Garg PK, Dixit SG, Krishan K, Kanchan T (2022) Computed tomographic evaluation of the acetabulum for age estimation in an Indian population using principal component analysis and regression models. Int J Legal Med 136:1637–1653. 10.1007/s00414-022-02856-4 35715653 10.1007/s00414-022-02856-4

[77] Macaluso PJ (2015) Test of the usefulness of acetabular size for stature estimation. Australian J Forensic Sci 47:239–249. 10.1080/00450618.2014.936896

[78] Macaluso PJ (2011) Sex discrimination from the acetabulum in a twentieth-century skeletal sample from France using digital photogrammetry. Homo 62:44–55. 10.1016/j.jchb.2010.11.001 21106196 10.1016/j.jchb.2010.11.001

[79] Arsuaga JL, Carretero JM (1994) Multivariate analysis of the sexual dimorphism of the hip bone in a modern human population and in early hominids. Am J Phys Anthropol 93:241–257. 10.1002/ajpa.1330930208 8147439 10.1002/ajpa.1330930208

[80] Nagesh KR, Kanchan T, Bastia BK (2007) Sexual dimorphism of acetabulum–pubis index in South-Indian population. Leg Med 9:305–308. 10.1016/j.legalmed.2007.05.003 10.1016/j.legalmed.2007.05.003 17616420

[81] Day MH, Pitcher-Wilmott RM (1975) Sexual differentiation in the innominate bone studied by multivariate analysis. Ann Hum Biol 2:143–151. 10.1080/03014467500000691 1052746 10.1080/03014467500000691

[82] Van Gerven DP (1972) The contribution of size and shape variation to patterns of sexual dimorphism of the human femur. Am J Phys Anthropol 37:49–60. 10.1002/ajpa.1330370107 5039737 10.1002/ajpa.1330370107

[83] Bidmos MA, Mazengenya P (2021) Accuracies of discriminant function equations for sex estimation using long bones of upper extremities. Int J Legal Med 135:1095–1102. 10.1007/s00414-020-02458-y 33179172 10.1007/s00414-020-02458-y

[84] Wankhede KP, Bardale RV, Chaudhari GR, Kamdi NY (2015) Determination of sex by discriminant function analysis of mandibles from a central Indian population. J Forensic Dent Sci 7:37–43. 10.4103/0975-1475.150304 25709318 10.4103/0975-1475.150304 PMC4330617

[85] Chatterjee PM, Krishan K, Singh R, Kanchan T (2020) Sex estimation from the femur using discriminant function analysis in a central Indian population. Med Sci Law 60:112–121. 10.1177/0025802419900576 32050848 10.1177/0025802419900576

[86] Bertsatos A, Athanasopoulou K, Chovalopoulou ME (2019) Estimating sex using discriminant analysis of mandibular measurements from a modern Greek sample. Egypt J Forensic Sci 9:25. 10.1186/s41935-019-0133-7

[87] Kemkes-Grottenthaler A (2005) Sex determination by discriminant analysis: an evaluation of the reliability of patella measurements. Forensic Sci Int 147:129–133. 10.1016/j.forsciint.2004.09.075 15567616 10.1016/j.forsciint.2004.09.075

[88] Renjith G, Mary DP, Soe K et al (2019) Sex estimation by discriminant function analysis using anatomical location of mental foramen. Forensic Sci Int: Rep 1:100018. 10.1016/j.fsir.2019.100018

[89] Krishan K, Chatterjee PM, Kanchan T, Kaur S, Baryah N, Singh RK (2016) A review of sex estimation techniques during examination of skeletal remains in forensic anthropology casework. Forensic Sci Int 261. 10.1016/j.forsciint.2016.02.007. :165.e1-165.e8 10.1016/j.forsciint.2016.02.007 26926105

[90] Kamath VG, Asif M, Shetty R, Avadhani R (2015) Binary logistic regression analysis of foramen magnum dimensions for sex determination. Anat Res Int 2015:e459428. 10.1155/2015/459428 10.1155/2015/459428 PMC4540976 26346917

[91] Verma R, Krishan K, Rani D, Kumar A, Sharma V, Shrestha R, Kanchan T (2020) Estimation of sex in forensic examinations using logistic regression and likelihood ratios. Forensic Sci Int: Rep 2:100118. 10.1016/j.fsir.2020.100118

[92] Attia MH, Aboulnoor BAES (2020) Tailored logistic regression models for sex estimation of unknown individuals using the published population data of the humeral epiphyses. Leg Med 45:101708. 10.1016/j.legalmed.2020.101708 10.1016/j.legalmed.2020.101708 32334366

[93] Zheng B, Zhong Y, Al-Worafi NA, Liu Y (2023) The dimensional and morphological assessment of the frontal sinus in sex estimation among different populations. Head Face Med 19:8. 10.1186/s13005-023-00355-4 36918984 10.1186/s13005-023-00355-4 PMC10012595

[94] Del Bove A, Veneziano A (2022) A generalised neural network model to estimate sex from cranial metric traits: a robust training and testing approach. Appl Sci 12:9285. 10.3390/app12189285

[95] Anic-Milosevic S, Medancic N, Calusic-Sarac M, Dumancic J, Brkic H (2023) Artificial neural network model for predicting sex using dental and orthodontic measurements. Korean J Orthod 53:194–204. 10.4041/kjod22.250 37226512 10.4041/kjod22.250 PMC10212777

[96] Bewes J, Low A, Morphett A, Pate FD, Henneberg M (2019) Artificial intelligence for sex determination of skeletal remains: application of a deep learning artificial neural network to human skulls. J Forensic Leg Med 62:40–43. 10.1016/j.jflm.2019.01.004 30639854 10.1016/j.jflm.2019.01.004

[97] Venema J, Peula D, Irurita J, Mesejo P (2023) Employing deep learning for sex estimation of adult individuals using 2D images of the humerus. Neural Comput Applic 35:5987–5998. 10.1007/s00521-022-07981-0

[98] Cao Y, Ma Y, Yang X et al (2022) Use of deep learning in forensic sex estimation of virtual pelvic models from the Han population. Forensic Sci Res 7:540–549. 10.1080/20961790.2021.2024369 36353321 10.1080/20961790.2021.2024369 PMC9639534

[99] Imaizumi K, Bermejo E, Taniguchi K et al (2020) Development of a sex estimation method for skulls using machine learning on three-dimensional shapes of skulls and skull parts. Foren Imag 22:200393. 10.1016/j.fri.2020.200393

[100] Mutlu GD, Asirdizer M, Kartal E, Keskin S, Mutlu I, Cemil GOYA (2024) Sex estimation from the hyoid bone measurements in an adult eastern Turkish population using 3D CT images, discriminant function analysis, support vector machines, and artificial neural networks. Leg Med 67:102383. 10.1016/j.legalmed.2023.102383 10.1016/j.legalmed.2023.102383 38159420

[101] Huseynov A, Zollikofer CP, Coudyzer W, Gascho D, Kellenberger C, Hinzpeter R, Ponce de León MS (2016) Developmental evidence for obstetric adaptation of the human female pelvis. P Natl Acad Sci USA 113:5227–5232. 10.1073/pnas.1517085113 10.1073/pnas.1517085113 PMC4868434 27114515

[102] Secgin Y, Oner Z, Turan MK, Oner S (2022) Gender prediction with the parameters obtained from pelvis computed tomography images and machine learning algorithms. J Anat Soc India 71(3):204–209

[103] Kartal E, Etli Y, Asirdizer M et al (2022) Sex estimation using foramen magnum measurements, discriminant analyses and artificial neural networks on an eastern Turkish population sample. Leg Med (Tokyo) 59:102143. 10.1016/j.legalmed.2022.102143 36084487 10.1016/j.legalmed.2022.102143

[104] Toneva D, Nikolova S, Agre G, Zlatareva D, Fileva N, Lazarov N (2024) Sex estimation based on mandibular measurements. Anthropol Anz 81. 10.1127/anthranz/2023/1733 10.1127/anthranz/2023/1733 37498011

[105] Demir U, Etli Y, Hekimoglu Y, Kartal E, Keskin S, Yavuz A, Asirdizer M (2022) Sex estimation from the clavicle using 3D reconstruction, discriminant analyses, and neural networks in an eastern Turkish population. Leg Med 56:102043. 10.1016/j.legalmed.2022.102043 10.1016/j.legalmed.2022.102043 35183842

[106] Mahfouz M, Badawi A, Merkl B et al (2007) Patella sex determination by 3D statistical shape models and nonlinear classifiers. Forensic Sci Int 173:161–170. 10.1016/j.forsciint.2007.02.024 17482786 10.1016/j.forsciint.2007.02.024

[107] Nikita E, Nikitas P (2020) On the use of machine learning algorithms in forensic anthropology. Leg Med 47:101771. 10.1016/j.legalmed.2020.101771 10.1016/j.legalmed.2020.101771 32795933

[108] Tague RG (1989) Variation in pelvic size between males and females. Am J Phys Anthropol 80:59–71. 10.1002/ajpa.1330800108 2801906 10.1002/ajpa.1330800108

[109] Kurki HK (2013) Skeletal variability in the pelvis and limb skeleton of humans: does stabilizing selection limit female pelvic variation? Am J Hum Biol 25:795–802. 10.1002/ajhb.22455 24123540 10.1002/ajhb.22455

[110] Lesciotto KM, Christensen AM (2024) The over-citation of Daubert in forensic anthropology. J Forensic Sci 69:9–17. 10.1111/1556-4029.15409 37855082 10.1111/1556-4029.15409

[111] Kelley MA (1979) Sex determination with fragmented skeletal remains. J Forensic Sci 24:154–158. 10.1520/JFS10802J 512598

[112] Stevens SS (1946) On the theory of scales of measurement. Science 103:677–680. 10.1126/science.103.2684.677 17750512

[113] Mestekova S, Bruzek J, Veleminska J, Chaumoitre K (2015) A test of the DSP sexing method on CT images from a modern French sample. J Forensic Sci 60(5):1295–1299. 10.1111/1556-4029.12817 26258990 10.1111/1556-4029.12817

